# Mechanical thrombectomy (MT) for acute ischemic stroke (AIS) in COVID-19 pandemic: a systematic review

**DOI:** 10.1186/s41983-021-00321-4

**Published:** 2021-06-02

**Authors:** Aditya Kurnianto, Dodik Tugasworo, Yovita Andhitara, Retnaningsih, Rahmi Ardhini, Jethro Budiman

**Affiliations:** Department of Neurology, Dr. Kariadi Hospital/Faculty of Medicine Diponegoro University, Semarang, Indonesia

**Keywords:** Acute ischemic stroke, COVID-19, Ischemic stroke, Mechanical thrombectomy

## Abstract

**Introduction:**

Coronavirus disease 2019 (COVID-19) is a disease caused by severe acute respiratory syndrome coronavirus 2 (SARS-CoV-2). Initially, COVID-19 is a disease that attacks the respiratory tract, but now the clinical manifestations of COVID-19 are various, including acute ischemic stroke (AIS). Emergency surgeries such as mechanical thrombectomy (MT) for AIS must be performed without any delay even during the COVID-19 pandemic, to reduce morbidity and mortality. Besides the focus on patient’s health, the safety of healthcare workers must also be considered. The aim of the study was to evaluate and summarize the scientific literature systematically to explore MT for AIS in the COVID-19 pandemic.

**Data synthesis:**

The independent reviewers searched the literature through 12 electronic databases, searching for articles fulfilling inclusion and exclusion criteria. The data from all included studies were presented in a summary table featuring key points of each study. The authors independently assessed the risk of bias of 15 included articles.

**Conclusion:**

Although MT procedure has been prolonged during the pandemic, clinical outcomes and procedure-related serious adverse events have remained unchanged during the COVID-19 pandemic. The screening process and the implementation of the PCS algorithm must be performed to reduce the spread of COVID-19 infection without threatening patient safety and clinical outcomes. The standard precaution of infection and the health assurance of healthcare workers and their families (including mental health) are also important factors that must be given special attention and consideration in the COVID-19 pandemic.

## Introduction

Coronavirus disease 2019 (COVID-19) is a disease caused by severe acute respiratory syndrome coronavirus 2 (SARS-CoV-2) [1–4]. Phylogenetic analysis showed that SARS-CoV-2 had 79.5% and 51% similarity with severe acute respiratory coronavirus syndrome (SARS-CoV) and Middle East respiratory coronavirus syndrome (MERS-CoV) [2, 5]. The first case of COVID-19 occurred in December 2019 in Wuhan, Hubei Province, China with sources of transmission linked to the fish market and several wild animals (birds, snakes, and bats) in Wuhan [1, 6, 7]. SARS-CoV-2 can spread through direct contact (droplet and transmission between humans) and indirect contact (airborne transmission and contaminated objects) [2]. SARS-CoV-2 has spread widely in more than 250 countries, until 12 March 2020 COVID-19 was established as a global pandemic by the World Health Organization (WHO) with a world fatality rate of 5.28% [8–11]. Until June 20, 2020, there were 8,745,570 cases worldwide with a total of 461,760 deaths [9].

Initially, COVID-19 is a disease that attacks the respiratory tract with angiotensin-converting enzyme 2 (ACE2) as the main receptor, but now the clinical manifestations of COVID-19 are various, including neurological disorders. A neurological disorder that needs special attention because of their morbidity and mortality that can be suppressed if treated in the golden period is acute stroke [12–18]. The cytopathic effect of the virus and dysregulation of the immune system can cause severe inflammation, including inflammatory cytokine storms that cause COVID-19-associated coagulopathy (CAC) or thrombosis, and can cause acute ischemic stroke (AIS) [13]. SARS-CoV-2 has also been shown to be associated with thromboembolic disease and hypercoagulability state through mechanisms of hypoxia, inflammation, and disseminated intravascular coagulation (DIC) [19]. Inflammation, CAC, and endothelial dysfunction can also increase the permeability of the blood brain barrier, so that the virus can enter the central nervous system (CNS) through transcellular, paracellular, and retrograde transport of axons through the sensory and olfactory nerves (lamina cribiform and olfactory bulb) [13, 16, 20]. The incidence of COVID-19 patients with stroke in China and Europe was about 2.5% to 6% [13, 21]. The most common type of stroke is ischemic stroke (84.6%), followed by central venous thrombosis (7.7%), and hemorrhagic stroke (7.7%) [16, 22].

In the beginning, mechanical thrombectomy (MT) for AIS had not shown a better outcome in comparison with conventional medical treatments. But now with the better understanding and procedure of MT, MT is reported to have a clear advantage for AIS over conventional medical treatments [23–25]. Currently, MT has been widely accepted as the first-line therapy for patients with emergent large vessel occlusion (LVO) stroke [23, 26, 27]. During the COVID-19 pandemic, many operations have been postponed or even canceled due to the need for SARS-CoV-2 infection screening and relocation of limited medical resources. Even though, emergency surgeries such as MT for AIS must be performed without any delay even during the pandemic, to reduce morbidity and mortality [28, 29]. Besides the focus on patient’s health, the safety of healthcare workers must also be considered in the COVID-19 pandemic. Protected code stroke is a term used during the COVID-19 pandemic to prioritize acute assessment and management of AIS patients and to provide safety and protection of healthcare workers and also patients [30]. Expert consensuses from Chinese and European Federation recommended that all patients including those receiving MT should undergo screening process (preoperative chest CT scan and multidisciplinary consultations to exclude COVID-19) and the PCS algorithm [29–32].

MT for AIS in COVID-19 pandemic have been reported and explained in various studies, but there was no systematic review about it. The aim of the study was to evaluate and summarize the scientific literature systematically to explore MT for AIS in the COVID-19 pandemic.

## Main text (review of the literature, results, discussion)

### Review of literature

#### Inclusion and exclusion criteria

Inclusion criteria in this systematic review were publication type was full-text articles discussing MT for AIS in COVID-19 pandemic and primary studies of every design (descriptive study, such as case report and case series; observational study such as cross-sectional, case-control, and cohort; and experimental study such as clinical trial); the language of publication was English; time of publication was in December 2019–December 2020; and objective, methodology, and outcome measure must explain MT for AIS in the COVID-19 pandemic. Exclusion criteria in this systematic review was confounding variables were related to outcome in MT for AIS in the COVID-19 pandemic.

#### Literature search

This systematic review was conducted according to the Cochrane handbook for systematic reviews and the guideline of preferred reporting items for systematic review and meta-analysis (PRISMA) [33, 34]. A systematic search literature was used in these electronic databases: Cambridge Core, Clinical Key, Ebsco, Emerald Insight, JSTOR, Medline, Nature, Proquest, PubMed, Science Direct, Scopus, and Springer Link. The search was conducted using the following keywords for title and abstract: (thrombectomy OR mechanical thrombectomy) AND (stroke OR ischemic stroke) AND (COVID-19 OR coronavirus OR SARS-CoV-2). The reference lists from retrieved literature were also examined to avoid missing any published data.

#### Data collection and analysis

Studies were selected for evaluation after two independent reviewers (AK and DT) had collected titles and abstracts identified in the electronic database. The results of the two independent reviewers were compared by a third independent reviewer (YA), and any differences of opinion were resolved by discussion. Full papers from potential studies were independently assessed by the investigators (R and RA). All studies selected for this systematic review were screened by two reviewers independently to validate the results (AK and JB). The data from all retrieved studies were presented in a summary table featuring key points of each study. The key points of each study were: first author, country, and year; study design; sample; outcome measure; and result.

#### Quality assessment

The lead author independently assessed quality assessment and risk of bias of each included study and discussed them with other authors. Quality assessment and risk of bias within studies were assessed using criteria developed by Hawker and colleagues 35, 36]. Ratings were concluded (very poor, poor, fair, good, and not applicable) across nine different categories: abstract and title; introduction and aim; method and data; sampling; data analysis; ethic and bias; result; generalisability; and implication and usefulness. The risk of bias potentially affecting the cumulative evidence across studies was determined by examining study methods, ethics committee approvals, study funding, and conflicts of interest [35, 36]. Newcastle–Ottawa scale for cohort study was also used to assess the methodological quality of prospective study; interpretation of total score was ≥ 7 points were included in good studies, 5–6 points were included in fair studies, < 5 points were included in poor studies. Newcastle-Ottawa scale adapted for cross-sectional study was used to assess the methodological quality of the cross-sectional study. Interpretation of total score was 9 to 10 points were considered in very good studies, 7 to 8 points were considered in good studies, 5 to 6 points were considered in satisfactory studies, and 0 to 4 points were considered in unsatisfactory studies [37–41]. The Joanna Briggs Institute (JBI) critical appraisal checklist was used to assess the methodological quality of the case report and case series [42–44].

### Results

#### Selection of articles for review

**Fig. 1:**
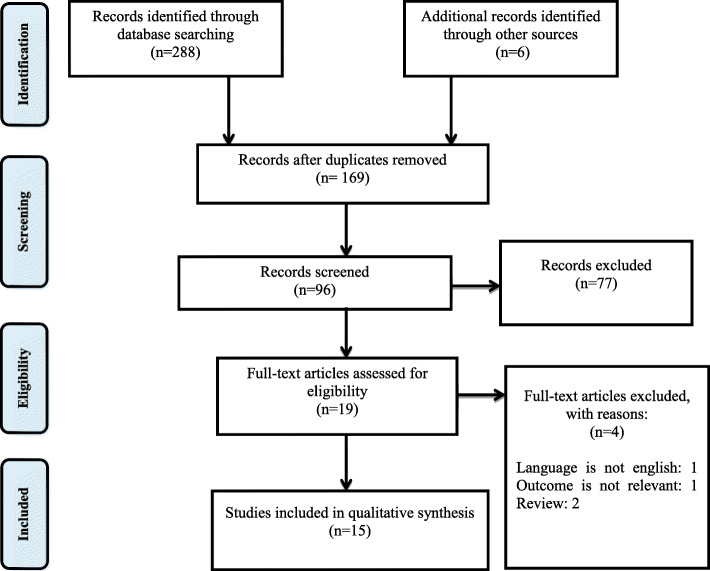
PRISMA flow diagram

Figure 1 summarized the identified, screened, and included articles for review. Initially, 288 peer-reviewed articles were identified from electronic databases and an additional 5 articles were identified through other sources (search engine). After removing duplicates, 168 articles remained for the title and abstract screening. Articles that did not meet the inclusion and exclusion criteria were not further screened. Eighteen articles were screened for eligibility of which 15 articles met all the inclusion criteria.

#### Assessment of study validity (quality assessment and risk of bias)

All eligible studies were associated with MT for AIS in the COVID-19 pandemic. Table 1 provided quality assessment and risk of bias by Hawker and colleagues, and all of the studies are fair and good. Table 2 provided quality scores for cohort study and the study got 7 points that were considered in good study. Table 3 provided quality scores for cross-sectional study and all of the studies got 5–8 points that were considered satisfactory and good studies. Tables 4 and 5 showed JBI critical appraisal checklist for case report and case series; all of the studies had overall appraisal in “included studies” for systematic review.

**Table 1:** Quality assessment and risk of bias by Hawker and colleagues

No.	First author, country	Abstract and title	Introduction and aim	Method and data	Sampling	Data analysis	Ethic and bias	Finding	Generalisability	Implication and usefulness
1.	Al Kasab S; North and South America, Europe [45]	Good	Good	Good	Good	Good	Good	Good	Good	Good
2.	Cox M, USA [46]	Good	Good	Fair	Fair	Fair	Good	Good	Good	Good
3.	Escalard E, France [47]	Good	Good	Good	Good	Fair	Good	Good	Good	Good
4.	Havenon A, USA [48]	Good	Good	Good	Good	Fair	Good	Good	Good	Good
5.	Kerleroux B, France [28]	Good	Good	Good	Good	Good	Good	Good	Good	Good
6.	Kwan J, UK [49]	Good	Good	Fair	Fair	Good	Good	Good	Good	Good
7.	Mansour OY, Egypt [50]	Good	Fair	Fair	Fair	Good	Fair	Good	Good	Good
8.	McConachie D, UK [51]	Good	Good	Good	Good	Good	Good	Good	Good	Good
9.	Pop R, France [52]	Good	Good	Good	Good	Good	Good	Good	Good	Good
10.	Tiedt, Germany [53]	Good	Good	Good	Good	Good	Good	Good	Good	Good
11.	Qureshi A I, USA [54]	Fair	Fair	Fair	Fair	Good	Fair	Fair	Fair	Fair
12.	Wang A, USA [19]	Good	Good	Fair	Fair	Good	Fair	Good	Good	Good
13.	Yaeger K A, USA [55]	Good	Good	Good	Fair	Good	Fair	Good	Good	Good
14.	Yang B, China [29]	Good	Good	Good	Good	Good	Good	Good	Good	Good
15.	Yeboah K, USA [56]	Fair	Fair	Fair	Fair	Good	Fair	Good	Good	Good

**Table 2:** Newcastle-Ottawa scale (cohort study)

No.	First author, year	Selection				Comparability	Outcome			Total
		1	2	3	4		1	2	3	
1.	Tiedt, Germany [53]	*	*	*	*		*	*	*	7

Maximum points for selection number 4, comparability, and outcome number 1 were 2

Selection: (1) representativeness of the sample, (2) sample size, (3) non-respondents, (4) risk factor measurement tool

Outcome: (1) assessment of the outcome, (2) statistical test

**Table 3:** Newcastle-Ottawa scale adapted for cross-sectional study

No.	First author, country	Selection				Comparability	Outcome		Total
		1	2	3	4		1	2	
1.	Al Kasab S; North and South America, Europe [45]	*	*	*	**	*	*	*	8
2.	Cox M, USA [46]	*	*		**		*	*	6
3.	Havenon Ad, USA [48]	*	*		**	*	*	*	7
4.	Kerleroux B, France [28]	*	*		**	*	*	*	7
5.	Kwan J, UK [49]	*	*		**	*	*	*	7
6.	McConachie D, UK [51]	*	*		**	*	*	*	7
7.	Pop R, France [52]	*	*		**	*	*	*	7
8.	Qureshi A I, USA [54]	*			*	*	*	*	5
9.	Yang B, China [29]	*	*	*	**	*	*	*	8

Maximum point for comparability was 2

Selection: (1) representativeness, (2) selection of non-exposed, (3) ascertainment of exposure, (4) demonstration that outcome was not present at the beginning

Outcome: (1) assessment of the outcome, (2) follow up long enough, (3) adequacy of follow up

**Table 4:** JBI Critical Appraisal Checklist for case report

No.	Major components	Masnour OY, Egypt [50]	Yeboah K, USA [56]
1.	Were patient’s demographic characteristics clearly described?	Yes	Yes
2.	Was the patient’s history clearly described and presented as a timeline?	Yes	Yes
3.	Was the current clinical condition of the patient on presentation clearly described?	Yes	Yes
4.	Were diagnostic tests or assessment methods and the results clearly described?	Yes	Yes
5.	Was the intervention(s) or treatment procedure(s) clearly described?	Yes	Yes
6.	Was the post-intervention clinical condition clearly described?	Yes	Yes
7.	Were adverse events (harms) or unanticipated events identified and described?	Yes	Yes
8.	Does the case report provide takeaway lessons?	Yes	Yes
	**Overall appraisal**	**Include**	**Include**

**Table 5:** JBI Critical Appraisal Checklist for case series

No.	Major components	Escalard E, France [47]	Wang A, USA [19]	Yaeger K A, USA [55]
1.	Were there clear criteria for inclusion in the case series?	Yes	Yes	Yes
2.	Was the condition measured in a standard, reliable way for all participants included in the case serie	Yes	Yes	Yes
3.	Were valid methods used for identification of the condition for all participants included in the case series?	Yes	Yes	Yes
4.	Did the case series have consecutive inclusion of participants?	Yes	Yes	Yes
5.	Did the case series have complete inclusion of participants?	Yes	Yes	Yes
6.	Was there clear reporting of the demographics of the participants in the study?	Yes	Yes	Yes
7.	Was there clear reporting of clinical information of the participants?	Yes	Yes	Yes
8.	Were the outcomes or follow up results of cases clearly reported?	Yes	Yes	Yes
9.	Was there clear reporting of the presenting site(s)/clinic(s) demographic information?	Yes	Yes	Yes
10.	Was statistical analysis appropriate?	Not applicable	Not applicable	Not applicable
	**Overall appraisal**	**Include**	**Include**	**Include**

#### Study characteristic

The study characteristics for the included studies could be seen in Table 5. There were one cohort study, eight cross-sectional studies, three case series studies, and one case report. The studies reported about patient characteristics (baseline characteristic, clinical presentation, laboratory examination, and radiology examination), MT procedure, clinical outcome, the care delays, and PCS of AIS in the COVID-19 pandemic (Table 6).

**Table 6:** Study characteristic

No.	First author, country	Study design	Sample (*n*)	Outcome measure	Result
1.	Al Kasab S; North and South America, Europe [45]	Cross-sectional	458	The effect of GA on mortality and discharge outcome	GA had longer door to reperfusion time (138 vs. 100 min, *p* < 0.001), higher mortality (RR: 1.871, *p*: 0.029), and lower functional outcome discharge (RR: 0.053, *p*: 0.015).
2.	Cox M, USA [46]	Cross-sectional	45	PCS in MT of AIS patients	The importance of PCS implementation and the use of PPE during MT.
3.	Escalard E, France [47]	Case series	10	Patient outcome	Successful MT was performed in 9 patients, none had good early neurological outcomes, and 6 patients died in the hospital.
4.	Havenon A, USA [48]	Cross-sectional	3145	Comparison of the outcome of MT in COVID-19 and non COVID-19	Mortality rate was increased significantly in AIS patients (treated with MT) with COVID-19 (29.8%) vs without COVID-19 (12.4%) (OR: 4.48, 95% CI: 3.02-6.165, *p* < 0.001). COVID-19 decreased a favorable hospital discharge (OR: 0.43, 95% CI: 0.3–0.61, *p* < 0.05).
5.	Kerleroux B, France [28]	Cross-sectional	1513	Comparison of MT in AIS patients before and during COVID-19 pandemic	There was a 21% reduction in MT case (OR: 0.79, 95%CI: 0.76-0.82, *p* < 0.001), significant delays between imaging to puncture time (mean 144.9 ± 86.8 vs. 126.2 ± 70.9 min, *p* < 0.001 in 2019) and imaging to in-transferred patients (mean 182.6 ± 82.0 vs. 153.25 ± 67 min, *p* < 0.001), compared with the same period in 2019. There was a significant negative correlation between the number of COVID-19 hospitalizations and the number of MT cases compared with the same period in 2019 (r: 0.51, *p*: 0.04).
6.	Kwan J, UK [49]	Cross-sectional	61	Comparison of MT in AIS patients before and during COVID-19 pandemic	During the COVID-19 pandemic, (a) MT rate was maintained at 20% of AIS and there was a non-significant 21% decrease in MT, referred from the external hospital (*p*: 0.067); (b) successful reperfusion rate was maintained at 81% and early neurological outcomes were not significantly different; (c) the use of general anesthesia reduced significantly from 85 to 32% (*p* < 0.05); and (d) time intervals from onset to arrival, puncture, and reperfusion were not significantly different, whereas internal delays for external referrals significantly increased for door to puncture time (48 [IQR 39–57] vs. 33 [IQR 27–44] minutes, *p*: 0.013) and door to reperfusion time (82.5 [IQR 61–110] vs. 60 [IQR 55–70] minutes, *p*: 0.018).
7.	Mansour OY, Egypt [50]	Case report	1	Patient outcome, PCS in MT of AIS patients	The NIHSS score decreased to 2 after reperfusion (from 14 before MT). The importance of PCS implementation and the use of PPE during MT.
8.	McConachie D, UK [51]	Cross-sectional	27	Comparison of MT in AIS patients before and during COVID-19 pandemic	Three centers did not perform MT, there was a 27.7% decrease of MT procedures in April 2020, and 22 centers reported delays of stroke care. 17 centers reported the reduction of training opportunities for specialist registrars and 14 centers reported the delay of development plans of MT service.
9.	Pop R, France [52]	Cross-sectional	122	Comparison of MT in AIS patients before and during COVID-19 pandemic	There were 39.6% reduction of stroke alerts and 27.6% decrease in MT procedures in March 2020, compared to the same period in 2019. There were no significant differences in time delays or clinical outcomes for patients treated by MT.
10.	Tiedt S, Germany [53]	Cohort	795	Comparison of MT in AIS patients before and during COVID-19 pandemic	There was prolonged door to groin time in 2020, compared with the same period in 2019 (47 min vs. 38 min, *p:* 0.005). Functional outcome of patients treated with MT in 2020 was not significantly different compared to patients treated in 2019 (*p* > 0.05).
11.	Qureshi A I, USA [54]	Cross-sectional	24	Comparison of MT in AIS patients before and during COVID-19 pandemic	There was a significant reduction of MT procedures in March 2020 (*p* < 0.05).
12.	Wang A, USA [19]	Case series	5	Patient demographic, laboratory value, MT technique, and clinical and outcome	4 patients with COVID-19 had AIS with occlusion in anterior circulation and 1 patient with occlusions in anterior and posterior circulation, the average of age was 52.8 years, and all patients had coagulation abnormalities. Stent-aspiration combination technique was performed in all patients with poor clinical outcomes.
13.	Yaeger K A, USA [55]	Case series	10	Patient outcome	Successful MT was performed in 9 patients with the NIHSS score decreased by an average of 7.7 points.
14.	Yang B, China [29]	Cross-sectional	55	Comparison of MT in AIS patients before and during COVID-19 pandemic	There was significant increase in door to puncture time (174 vs. 125.5 min, *p*: 0.002) and door to reperfusion time (213 vs. 172 min, *p*: 0.047) in the COVID-19 pandemic, compared with the same period in 2019. The rate of successful MT was not significantly different between the two groups (85.7% (*n* = 18) vs. 88.2% (*n* = 30), OR 0.971, 95% CI: 0.785–1.203; *p*: 1.000).
15.	Yeboah K, USA [56]	Case report	1	Patient outcome, PCS in MT of AIS patients	The NIHSS score decreased to 5 after reperfusion (from 14 before reperfusion) and reduced to 0 in day 2 post-reperfusion. The importance of PCS implementation and the use of PPE during MT.

### Discussion

#### MT for AIS in COVID-19 pandemic

There are some guidelines related to MT for AIS in the COVID-19 pandemic but there is no systematic review about it. The differences about MT for AIS in COVID-19 pandemic and non-pandemic are about patient characteristics (baseline characteristic, clinical presentation, laboratory examination, and radiology examination), MT procedure, clinical outcome, the care delays, and PCS. The differences in patient characteristics were not discussed in this systematic review because they were more related to stroke in COVID-19 generally. All of the AIS types were LVO because MT was frequently used in LVO case [23, 26, 27].

Kerleroux and colleagues (2020) reported about 21% decrease in MT cases in the first month of the COVID-19 pandemic in French [28]. Kwan and colleagues (2020) reported a decrease of 21% in MT procedure from the external referral hospital in UK [49]. McConachie and colleagues (2020) reported about 27.7% decrease of MT procedure in April 2020 compared with the first 3 months of 2020 in the UK [51]. Qureshi and colleagues (2020) reported significant reduction of MT procedure in the USA [57]. The most possible reason was the strict guidelines of stroke care centers to perform MT in eligible patients. Patients with indications outside of strict guidelines may have not been accepted for MT [28, 49]. Other plausible reasons were an increase in healthy lifestyles during the COVID-19 pandemic (reduced the incidence of stroke), the strict PCS and screening procedure, or it could be due to the patient's reluctance to come to the hospital/emergency room (especially in “mild” symptoms of stroke) for fear of being exposed to the COVID-19. It is important to explain and educate the public that this is not a problem because the hospital has taken standard precautions to ensure that patients and medical personnel are protected and the hospital remains the best and safe place to provide appropriate treatment for emergency cases such as AIS care. Hospital administration must also ensure patient safety through appropriate standard precautions and the use of personal protective equipment (PPE) [10, 58]. PCS is the modified of regular code stroke during the COVID-19 pandemic to provide an additional layer of protection (including PPE) for patients and medical personnel who were engaged in triage, rapid assessment, COVID-19 screening, and treatment of patients [30]. Cox and colleagues (2020), Mansour and colleagues (2020), Yaeger and colleagues (2020), and Yeboah and colleagues (2020) and reported about the importance of healthcare worker to follow PCS algorithm and the use of appropriate PPE to reduce the risk of COVID-19 infection [46, 50, 55, 56].

Pop and colleagues (2020) reported that there were 39.6% reduction of stroke alerts and 27.6% decrease in MT procedure [52]. Yang and colleagues (2020) reported about the prolonged door to puncture time (by 48.5 min, *p*: 0.002) and door to reperfusion time (by 41 min, *p*: 0.047) [29]. Kerleroux and colleagues (2020) reported the delays between imaging to groin time (by 29 min, *p* < 0.001) [28]. Tiedt and colleagues (2020) reported about the longer of door to puncture time in patients (47 min vs. 38 min, *p*: 0.005) [53]. The plausible reasons were the saturation of health medical resources as a result of the COVID-19 pandemic; screening process and the extra standard and isolation precautions applied in PCS; social distancing of each individual; and also the fear of medical worker’s, patient’s, or family’s fear of COVID-19 contamination risk at the hospital [28, 29, 52, 53]. In some research, these care delays did not impact the short-term outcomes. Although there was no significant difference to short-term outcome (follow up with the national institutes of health stroke scale/NIHSS in day 1–3 after MT), there were not long-term follow-up (such as 90 days of the modified rankin scale/mRS) to evaluate the long-term efficacy [28, 29, 49, 50].

MT technique used in COVID-19 pandemic is various related to the operator (direct aspiration first-pass technique/ADAPT, stent retrieval, or stent-aspiration combination/solumbra technique) [29]. In Wang A and colleagues (2020), the operator used solumbra technique [29]. Solumbra technique is safe technique and increases the rate of successful reperfusion [24, 29]. The difficulties of the MT process in COVID-19 pandemic were re-occlusion of the vessel soon after reperfusion (related to systemic hypercoagulability, microvascular thrombo-inflammation, and endotheliitis), low rate of fragmentation, and distal embolization (due to clot composition of reddish clot-suggestive high red blood cell content and neutrophil extracellular traps) [19, 47]. Yang and colleagues (2020) reported about the reduction of puncture to reperfusion time (from 40.5 to 32 min) without the effect of patient safety and successful reperfusion rates during the COVID-19 pandemic. A series of ways were performed to reduce the time of the MT procedure, including performing the entire procedure by an experienced neurointerventional specialist and reducing angiography processes based on good CT angiography images. Other possible reasons were the use of local anesthesia and first-line ADAPT strategy [29]. Sedation and anesthesia choice for MT in COVID-19 pandemic present its own challenges and limited published data are available at this time to guide appropriate decisions and conclusions. Al Kasab and colleagues (2020) reported that general anesthesia was associated with poor functional outcome and mortality in COVID-19 patients [45]. General anesthesia with intubation carries the appeal of a closed respiratory circuit and the load of viral particles in the environment is decreased. However, bag-mask ventilation and intubation itself are highly aerosol-generating procedures. The negative pressure of environment and room, the minimum number of staff with full PPE, and the use of video laryngoscopy can be used to reduce the risk of COVID-19 infection. The use of conscious sedation when possible (such as sufficient oxygenation) avoids this intense aerosolization. The appropriate MT technique and anesthesia choice is still being debated and further research is needed [19, 29, 49, 50, 59, 60].

Clinical outcomes in patients with stroke and COVID-19 who undergo MT are various in some studies. Escalard and colleagues (2020) and Wang and colleagues (2020) reported about the poor clinical outcome; otherwise, Mansour and colleagues (2020), Yaeger and colleagues (2020), Yang and colleagues (2020), and Yeboah and colleagues (2020) reported about the good clinical outcome; while Kwan and colleagues (2020) and Tiedt and colleagues (2020) reported about the similar clinical outcome in pre-COVID-19 vs. COVID-19 periods [19, 29, 47, 50, 55, 56]. The difference in patient characteristics affected on this difference and need further research with similar characteristics on the global scale. Havenon and colleagues (2020) reported an increased risk of death in patients who undergo MT with comorbid COVID-19 [48]. The systemic complication (including acute respiratory failure, acute renal failure, and coagulopathy), and the delay of diagnosis and treatment in COVID-19 patient were the likely explanation of it [13, 19, 48].

#### Strength and limitation of the study

This systematic review involved studies that reported 15 studies related to MT for AIS in the COVID-19 pandemic. Most of the studies were analytical observational studies (eight) studies and the majority of the studies discussed the comparison of MT in AIS patients before and during the COVID-19 pandemic.

The limitation of the study was the variance of the demography, limited follow-up time, confounding variable in each study (there were confounding variables that cannot be controlled in human subjects), and also the limitation of study type (only an observational study, and the cohort study was only one).

#### Future implication

The current systematic review can be a scientific reading, material, and consideration to physician, researcher, and all of the readers related to MT for AIS in the COVID-19 pandemic. Further research is needed for the selection of appropriate MT technique and anesthesia choice, and also the evaluation of long-term follow-up related to MT for AIS in the COVID-19 pandemic.

## Conclusion

Although MT procedure has been prolonged during the pandemic, clinical outcomes and procedure-related serious adverse events have remained unchanged during the COVID-19 pandemic. The screening process and the implementation of the PCS algorithm must be performed to reduce the spread of COVID-19 infection without threatening patient safety and clinical outcomes. The standard precaution of infection and the health assurance of healthcare workers and their families (including mental health) are also important factors that must be given special attention and consideration in the COVID-19 pandemic.

## Data Availability

All data generated or analyzed during this study are included in this published article.

## Abbreviations

95% CI: 95% confidence interval
ACE2: Angiotensin-converting enzyme 2
AIS: Acute ischemic stroke
CAC: COVID-19 associated coagulopathy
CNS: Central nervous system
COVID-19: Coronavirus disease 2019
GA: General anesthesia
IQR: Interquartile range
LVO: Large vessel occlusion
min: Minutes
MERS-CoV: Middle East respiratory coronavirus syndrome
MT: Mechanical thrombectomy
NIHSS: The National Institutes of Health Stroke Scale
OR: Odds ratio
p: Probability
PCS: Protected code stroke
PPE: Personal protective equipment
PRISMA: Preferred reporting items for systematic review and meta-analysis
r: Correlation coefficient (correlation test)
RR: Relative risk
SARS-CoV: Severe acute respiratory syndrome coronavirus
SARS-CoV-2: Severe acute respiratory syndrome coronavirus 2
